# Effect of Stabilized Martensite on the Long-Term Performance of Superelastic NiTi Endodontic Files

**DOI:** 10.3390/ma16114089

**Published:** 2023-05-31

**Authors:** Patricia Sánchez, Benedetta Vidi, Jesús Mena-Alvarez, Javier Gil, Cristina Rico, Juan Manuel Aragoneses

**Affiliations:** 1 Bioengineering Institute of Technology, Facultad de Medicina y Ciencias de la Salud, Universitat Internacional de Catalunya, 08195 Sant Cugat del Vallés, Spain; patricia.sanchez@uax.es; 2 Programa de Doctorado Ciencia de la Salud, Universidad de Alcalá, Pza. San Diego s/n, 28801 Alcalá de Henares, Spain; benedetta.vidi@uax.es; 3 Faculty of Dentistry, Universidad Alfonso X El Sabio, C. de Emilio Muñoz, 13, 28691 Madrid, Spain; cromaric@uax.es (C.R.); jaraglam@uax.es (J.M.A.); 4 Department of Dental Research, Federico Henriquez y Carvajal University, Santo Domingo 10106, Dominican Republic

**Keywords:** stabilized martensite, fatigue, superelasticity, NiTi, endodontics files

## Abstract

One of the most used rotary files in endodontics is NiTi files due to their superelastic properties. This property means that this instrument has extraordinary flexion that can adapt to large angles inside the tooth canals. However, these files lose their superelasticity and fracture during use. The aim of this work is to determine the cause of fracture of the endodontic rotary files. For this purpose, 30 NiTi F6 SkyTaper^®^ files (Komet, Germany) were used. Their chemical composition was determined by X-ray microanalysis, and their microstructure was determined by optical microscopy. Successive drillings were carried out with artificial tooth molds at 30, 45, and 70°. These tests were carried out at a temperature of 37 °C with a constant load controlled by a high sensitivity dynamometer of 5.5 N, and every five cycles were lubricated with an aqueous solution of sodium hypochlorite. The cycles to fracture were determined, and the surfaces were observed by scanning electron microscopy. Transformation (austenite to martensite) and retransformation (martensite to austenite) temperatures and enthalpies were determined by Differential Scanning Calorimeter at different endodontic cycles. The results showed an original austenitic phase with a M_s_ temperature of 15 °C and A_f_ of 7 °C. Both temperatures increase with endodontic cycling, indicating that martensite forms at higher temperatures, and the temperature must be increased with cycling to retransform it to austenite. This fact indicates the stabilization of martensite with cycling, which is confirmed by the decrease in both transformation and retransformation enthalpies. The martensite is stabilized in the structure due to defects and does not retransform. This stabilized martensite has no superelasticity and, therefore, fractures prematurely. It has been possible to observe the stabilized martensite by studying the fractography, observing that the mechanism is by fatigue. The results showed that the files fracture earlier the greater the angle applied (for the tests at 70° at 280 s, at 45° at 385 s, and at 30° at 1200 s). As the angle increases, there is an increase in mechanical stress, and, therefore, the martensite stabilizes at lower cycles. To destabilize the martensite, a heat treatment can be carried out at 500 °C for 20 min, and the files recovers all its superelasticity.

## 1. Introduction

NiTi alloys are commonly used in endodontics because they combine superelasticity with excellent corrosion resistance, wear properties, and good biocompatibility [1,2,3,4]. The superelasticity is especially useful in endodontics because the alloy presents an important elastic deformation, and this allows large flexures in the dental canals [5,6,7]. This fact allows the instruments used in endodontics to be adapted to the canals of the teeth.

Superelasticity results from a stress-induced phase transformation. The formation of a martensitic phase in the alloy is initiated by cooling the material below M_s_, which can be defined as the temperature at which the martensitic transformation begins. M_f_ is the temperature at which the martensitic transformation ends. This transformation is reversible, being A_s_ the temperature at which the reverse austenitic transformation (Martensite → Austenite) starts upon heating, and A_f_ the temperature at the end of the reverse austenitic transformation [8,9,10]. The austenitic phase has an elastic deformation of approximately 15%, in contrast to conventional metals, whose elastic deformation is usually 0.2%. This excellent elastic behavior is due to the fact that austenite when subjected to stress, produces a stress-induced martensitic transformation. When this stress is relaxed, the martensite is elastically retransformed to austenite. However, the martensitic phase, if the stress continues to be applied, causes plastic deformation until fracture [10,11,12].

Despite the advantages of superelasticity in NiTi alloy, instrument fracture remains a major clinical concern. Fractures of endodontic instruments can occur in two ways: torsional fracture and bending fracture/fatigue [13]. The first occurs when the tip of the instrument remains locked in the root canal while its shaft continues to rotate. This situation exceeds the elasticity limit of the instrument, leading to plastic deformation and, finally, fracture. The other type of fracture is caused by stress and fatigue of the metal, resulting in a flexural fracture, and occurs mainly in narrow and curved canals [14]. 

Strategies have been implemented to increase the efficiency and safety of NiTi rotary files, including improvements in the manufacturing process or the use of new alloys that provide superior properties [15,16]. The mechanical properties and behavior of the NiTi alloy vary according to its composition and thermal/mechanical treatment during manufacturing [13,17,18]. Among these improvement treatments, we can find electropolishing [19], M-wire alloys [20], CM-Wire [21], R-Phase [22], Blue-Wire [23], Gold-Wire [24], Max-Wire [25], Fire-Wire [26], among others. Thermomechanical treatment of NiTi alloy allows a change in the phase composition leading to the appearance of martensite or R-phase under clinical conditions. Whilst M-Wire and R-phase instruments maintain an austenitic state, CM Wire, and the Gold and Blue heat-treated instruments, is composed of substantial amounts of martensite [22]. The austenitic instruments possess superelastic properties and reveal high torque values at fracture. Thus, these files are appropriate to shape straight or slightly curved root canals. Additionally, the use of austenitic alloy in pathfinding instruments may compensate for the decreased torque resistance caused by the smaller diameter of these files.

The aim of this study is to determine the fracture mechanisms of NiTi endodontic rotary files and how they influence the angles of application on the tooth. In other words, the reasons why over time, the endodontic rotary files lose their superelasticity until they fracture. Another objective would be to determine a treatment that could restore superelasticity before fracture to extend the life of endodontic burs.

The hypothesis of the contribution is that the austenite phase transforms to martensite and causes a loss of superelasticity. As the angle of application increases, the bending stresses are higher and will fracture earlier. It is possible to design a heat treatment that restores the austenite in the burs and gives back superelastic properties.

## 2. Materials and Methods

### 2.1. Materials

NiTi endodontic rotary files near equiatomic compositions were studied. The chemical compositions were determined by means of X-Ray microanalysis (Oxford Instrument X10, Oxford, UK) being: 51.2% Ni and 48.8% Ti (in atomic percentage).

**Figure 1:**
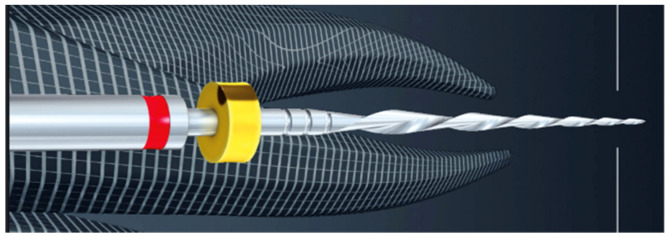
F6 SkyTiper@ 25.06 endodontic rotary files used.

The endodontic files used are of the brand F6 SkyTaper^®^ files (Komet Lemgo, Nordrhein Westfalen, Germany) (Figure 1) and are placed on a motor X-Smart® of Dentsply Sirona (Charlotte, NC, USA) with a speed of 300 rpm and torque of 2.2 Ncm.

**Figure 2:**
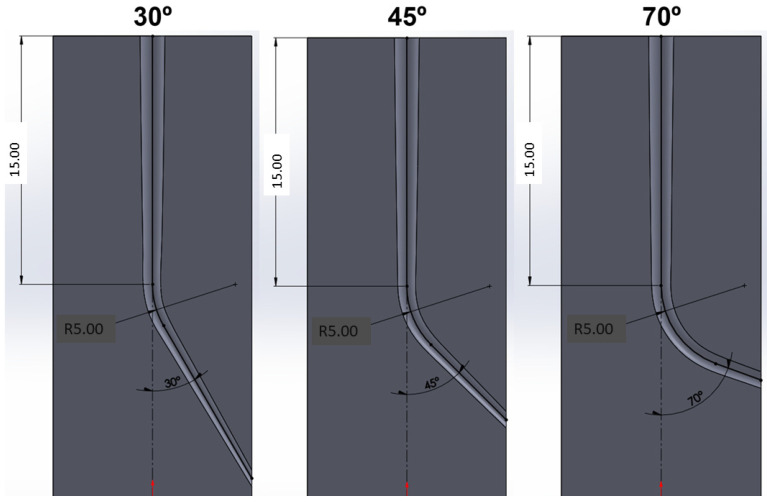
Scheme of the different molds for the endodontics tests. 30°, 45°, and 70° angles have been used, which are common angles in the clinic [27].

Endodontic cycles are performed on molds made of polyamide with properties very similar to the natural tooth obtained by 3D printing with canals with angles of 30, 45, and 70°. These are the most common tooth canal angles in patients [27]. The file force on the mold was 5.5 N, and they were lubricated every 5 cycles with an aqueous solution of 5% sodium hypochlorite. For the determination of the force to which the file is subjected on the mold, different measurements were made on 25 clinicians of the Clínica Universitaria Odontológica Alfonso X el Sabio, obtaining a mean value of 5.5 N with a standard deviation of 2.8 N. The high precision dynamometer was Adamel Lombhragy (X1234, Lyon, France) and is adjusted to the hand of the clinician who will do the tests with maximum force control adjusted. The diagrams of the molds used can be seen in Figure 2. 

For the test, it was important that the lubrication of the file was as similar as possible to what occurs in the clinic, and for this purpose, the instrument was soaked in the aqueous solution, which was at 37 °C. This meant that the variations in the temperature of the file were negligible, as this could affect the phases present in the file if they were overheated.

Cycles of 60, 150, and 200 s, and even fractures were made. Once the drills had reached these cycles, the transformation and retransformation temperatures and enthalpies were determined by calorimetry. The fracture files were also observed by microscopy to determine the phases present and the fractography.

### 2.2. Calorimetric Tests

Five samples for each endodontic rotary file and for each thermal treatment were analyzed, all of them 25.0 mm long and 0.46 mm in diameter. The transformation temperatures were measured by means of a calorimeter Melcor S 10. The calorimetric system used was based on a flow calorimeter, which measured differential signals (ΔT) by means of thermocouples batteries. The temperature was measured by means of a standard Pt-100 probe. All signals were digitalized through a multichannel recorder and linked to a microcomputer. M_s_ and A_s_ transformation temperatures occur when there is a sudden increment in the calorimetric signal. In the same way, the final temperatures, M_f_ and A_f_, were determined when the calorimetric signal returned to the baseline [28]. The enthalpies were calculated as the area of the transformation and retransformation curves. For this calculation, the samples were weighed on a precision balance (sensitivity of 0.00001 g) (Sartorius 298-s, Barcelona, Spain).

### 2.3. Microstructures

The samples were polished metallographically with diamond paste from 5 mm to 0.1 mm and etched with an acidic mixture (17 mL of HF + 33 mL of HNO_3_ + 50 mL of H_2_O). The microstructures were observed using optical and scanning (SEM) using a JEOL 6400 (JEOL, Tokyo, Japan) and JEOL 1200 EXII. Microscopy was equipped with a dispersive energy x-ray microanalysis (Oxford Instruments, Oxford, UK), which was used for determining the chemical composition.

### 2.4. Heat Treatments

Heat treatments were carried out at different heat treatment temperatures in an electric furnace (Hobersal, Caldes de Montibui, Spain) at 300, 400, and 500 °C for 20 min in an attempt to de-anneal the martensite. Subsequently, they were quenched in water at room temperature, and the transformation temperatures were determined by calorimetry.

### 2.5. Statistical Analysis

The data was statistically analyzed using Student’s *t*-tests, one-way ANOVA tables, and Turkey’s multiple comparison tests in order to evaluate any statistically significant differences between the sample groups. The differences were considered significant when *p* < 0.05. All statistical analyses were performed with MinitabTM software (Minitab release 13.0, Minitab Inc., State College, PA, USA).

## 3. Results

**Figure 3:**
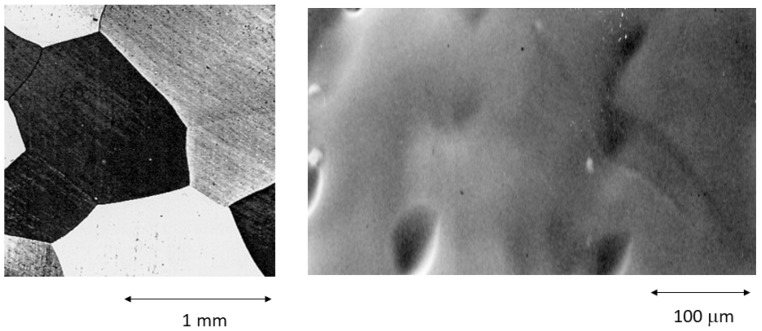
Austenitic observed at different magnifications as-received NiTi endodontic rotary file. High grain size and the absence of impurities, twins, or other defects can be seen.

The metallography of the original file corresponds to the austenitic phase, as can be seen in Figure 3. This microstructure is as expected since the transformation temperatures obtained by calorimetry were M_s_ = 15 °C and M_f_ = 5 °C, and for the transformation from martensite to austenite, they were A_s_ = −3 °C and A_f_ = 7 °C. The transformation enthalpies were 4342 J/g, and for the retransformation, −4312 J/g.

**Figure 4:**
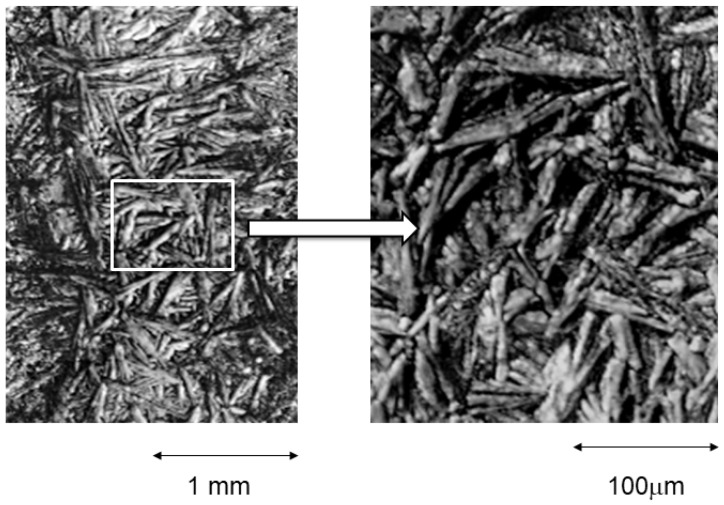
Martensite stabilized by stress after the fracture of the endodontic rotary file after 1200 s of use. The stress-induced martensite plates do not have an orientation because the torsional stresses do not have a unique direction.

The microstructure of the fractured file can be seen in Figure 4, where the martensitic phase can be seen. No preferential direction is observed since the stresses are not uniaxial [29,30].

Table 1 shows the fracture cycles of the files as a function of the application angle. It can be observed that as the angle increases, the fracture cycles are lower because the drill exerts higher bending stresses than the rest. In the fractured samples, it was not possible to obtain the temperatures or the transformation enthalpies since there is no martensitic transformation when cooling the sample or retransformation to austenite when heating up to 250 °C, which is the limit of the calorimeter used. This is due to the fact that the martensite that can be observed in Figure 4 is anchored in defects and is stable when heating to 250 °C; therefore, the file loses the superelastic properties of the austenitic phase [31].

The endodontic tests were stopped at different cycles to see the evolution of the transformation temperatures, and the transformation enthalpy. In Table 2, it can be seen that there is a statistically significant increase in the M_s_ and A_f_ temperatures with respect to the original burs. Table 2 also shows that both the transformation and retransformation enthalpies decrease as the file is cycled. This enthalpy indicates the value of heat absorbed for austenite to martensite trans-formation (endothermic) and heat expelled for martensite to austenite retransformation (exothermic). This reduction in enthalpies indicates that the amount of transformed or retransformed material is lower, indicating the presence of a phase (stabilized martensite) not susceptible to transformation [32,33,34]. 

**Figure 5:**
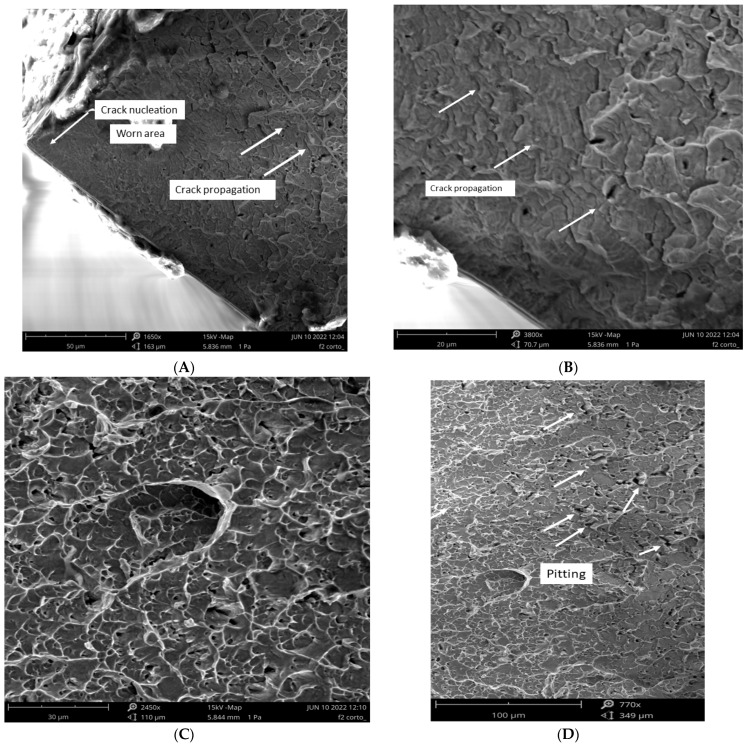
Fractography of the endodontic rotary file after 1250 cycles. (**A**) Location of crack initiation on the file surface. (**B**) Crack propagation towards the inside of the file. The striations indicate the direction of crack propagation. (**C**) Ductile fracture of the cutter with significant plastic deformation. (**D**) Corrosion pitting on the fracture surface. The pitting is observed in the direction of crack advancement, as these are the areas of highest energy.

Fractography studies show a rotary fatigue fracture. Figure 5A shows the beginning of the fracture, and we can indicate the crack initiation zone that is generated on the surface of the milling cutter. It can be seen how the first zone is with great deformation, showing a worn surface. This wear occurs because the cracked surfaces rub against each other in the milling process. This zone is followed by the crack propagation zone, which in the samples we have observed, is generated at about 500 μm from the place of crack nucleation. This zone can be seen in Figure 5B, where the crack advance marks can be seen, indicating the direction of propagation. The crack propagation causes the effective area that supports the milling stresses to become smaller and smaller until it cannot support the stresses, and the final fracture occurs. Figure 5C shows the ductile fracture of NiTi. In the different fractographies, pitting can be seen, which occurs because the NiTi is subjected to high mechanical stress on this fracture surface in an environment of lubrication with saline solution and therefore generates characteristic pitting due to its rounded shape. Figure 5D shows the area most exposed to this electrochemical corrosion since it is the initial area that has suffered great wear and therefore stored more residual stress and, in turn, more contact time with the aggressive solution. In this zone, it can be seen that pitting occurs around the grain boundaries [35,36]. This is due to the fact that these are the areas with the highest internal energy of the metal. It is well known that the places most susceptible to pitting are those with the highest mechanical stress, and these correspond to the grain boundaries of the areas with the highest wear [37].

The heat treatments were carried out with the purpose of eliminating the slip and helicoidal dislocations and twins produced by clinical practice. In this way, the martensite is released and can be retransformed to austenite. The samples where the heat treatments were performed were the fractures of the drills to ensure the highest number of defects. The heat treatments for 20 min at different temperatures gave the results shown in Table 3.

## 4. Discussion

The microstructure of the endodontic rotary file in its original state is completely austenitic, which is the phase that provides superelasticity to the instruments. This fact is corroborated by the M_s_ temperatures, which are lower than the oral temperature of 37 °C. When mechanical stress is applied when the bur is introduced into the tooth canal for endodontic treatment, a new phase is generated, which is stress-induced martensite. This phase returns to the austenitic phase when the stress disappears, returning to its original position and to its original phase, which is austenite. This property is called superelasticity [38,39].

With the passing of the endodontic rotary files cycles and the successive applications of stress, some stress-induced martensitic plates become anchored in the defects, such as dislocations or grain boundaries, and do not retransform to the original austenitic phase [40,41,42]. This fact causes a loss of the superelasticity property. The appearance of these martensitic plates, which are called stabilized, do not present an elastic deformation higher than 0.3% and, therefore, gradually lose the superelasticity of the drill and, therefore, its toughness [41,43].

The appearance of the stabilized martensite can be confirmed by the increase in the M_s_ temperature; that is to say, as the cycles pass, the martensite is generated more easily since the stabilized martensitic plates act as nucleation points of the new plates. It is for this reason that the M_s_ temperature increases, reaching values higher than 37 °C—the temperature of the human body-. Consequently, the file no longer shows superelasticity, but rather the instrument behaves in a plastic manner since the microstructure has a high content of non-superelastic stabilized martensite phase. As the proportion of stabilized martensite increases, the file becomes more and more brittle until it breaks [44].

The same fact is verified with the increase in the A_f_ temperature, i.e., the temperature returns to the austenitic phase due to heating. Calorimetry studies show that fractured files present temperatures A_f_ higher than the initial one since the system tries to heat up more to achieve the retransformation of martensite to the austenitic phase. The temperature increase is necessary to unanchor some plates that have been retained by the defects, and that is why more energy input is needed to achieve austenite. Some plates are so stabilized that retransformation is no longer achieved, but their stability does not allow, not even by the effect of heat, the reappearance of the austenitic phase, which is the superelastic phase [45].

This percentage of stabilized martensitic phase is confirmed by the decrease in the enthalpy of transformation from the austenitic phase to the martensitic phase and the enthalpy of retransformation from the martensitic phase to the austenitic phase. This decrease in the transformation enthalpies both in the endothermic and exothermic part is due to the fact that with the cycles, there is more and more stabilized martensite that does not transform with the temperatures offered by calorimetry [46,47,48]. There is no absorption of energy or transfer of transformation or retransformation energy for a phase—stabilized martensite—which is stable and will not transform [48].

Therefore, from the calorimetric results, we can determine that the cause of the endodontic rotary files failures is the embrittlement of the instruments due to the appearance of stabilized martensite, which causes the loss of the superelastic capacity of the original austenitic phase of the NiTi endodontic files.

From the results in Table 2, it can be seen that the files subjected to the higher angles break earlier, and the differences in the transformation temperatures at the same number of cycling times can be seen. It can be seen that the temperature changes are greater in the files that have worked at angles of 70°. By having to apply greater bending to the drilling, stress-induced martensite plates are formed more easily, and this leads to the appearance of a greater number of linear defects in the metal matrix of the milling cutters and, therefore, the stabilization of the martensite occurs more easily, and therefore, the milling cutters fracture earlier.

Annealing heat treatments at 300 °C are insufficient to cause the stabilized martensite to transform to austenite since calorimetry does not provide transformation curves. It is at 400 °C when the martensite destabilization begins as transformation temperatures start to be obtained, but still far from the original transformation temperatures. This fact can be verified by the fact that the values of the enthalpies are approximately 50% of the original ones, which indicates that the microstructure still has stabilized martensite that does not transform. It is at a temperature of 500 °C for 20 min that the drill recovers the original transformation temperatures, and therefore the material recovers its superelasticity. The heat treatment acts as a process of restoration of its microstructure, of the crystalline defects (vacancies and dislocations) generated by the use of the file. In other words, the material recovers its original phase—the austenite—its superelastic capacity without crystalline defects generated by the drilling process. These results are important for the clinical use of endodontic rotary files since heat treatments, such as the one performed in this work, would regenerate the NiTi and could extend the life of the files [49,50,51].

This research confirms the causes of fractures in endodontic rotary files. We have been able to demonstrate that the stabilized martensite that is formed with the endodontic cycles causes the gradual loss of superelasticity. We have also been able to determine the thermal treatments that prevent this loss of superelasticity, and therefore, heating at 500 °C for 20 min would allow the burs to have their initial properties. The work has limitations since the molds are made of polymeric material with properties very similar to the tooth, and the lubrication is carried out simulating that which occurs in clinical practice, and the forces of 5.5 N are an average value. Therefore, the experimentation may deviate slightly from clinical reality, but it is sufficient to demonstrate the causes of fracture of superelastic NiTi endodontic rotary files. Therefore, the hypothesis of the work has been confirmed by the microstructural and calorimetric experimentation of the files.

A limitation of the present research is that we have only carried out the studies with one milling cutter design and a given chemical composition. Design changes could change the results as the shear capacity and stress distribution in NiTi are modified [22,25,26]. Chemical composition performs a very important role in the behavior, although most of the burs used in endodontics have chemical compositions that are equal or very close, as in this case. The variation in the chemical composition will modify the transformation temperatures and, therefore, the cycles for the formation of stabilized martensite. The reason why Ni and Ti equiatomic alloys are used is that for these compositions’ austenite is obtained at room temperature and at a body temperature of 37 °C [52,53]. Increases in titanium can lead to the appearance of precipitates in fatigue or high-stress processes, which cause the loss of superelastic properties, similar to what happens with stabilized martensite. In the same way, nickel-rich precipitates have been obtained for nickel-rich alloys that cause brittle fractures [54].

## 5. Conclusions

From the experimental results, it has been shown that the higher the angle of application of the endodontic files, the number of cycles to fracture decrease. It has been observed that as we increase the cycles at the different angles studied, the presence of stabilized martensite in the NiTi microstructure increases until fracture. The presence of stabilized martensite has been verified by the increases in the temperatures M_s_ and A_f_ and by the decrease in the values of the transformation and retransformation enthalpies. Stabilization of the martensite causes the files to lose their superelastic properties, and thus, fracture occurs prematurely. Heat treatment at 500° for 20 min causes the transformation from stabilized martensite to austenite, and the file returns to superelastic characteristics. This fact must be considered to increase the life of the endodontic files. 

## Figures and Tables

**Table 1:** Cycles to fracture for the F6 SkyTaper^®^ file at different angles.

Angle	Cycles (s)
30	1250
45	760
70	250

**Table 2:** Transformation and retransformation temperatures and enthalpies for different cycles at different angles.

Angle	Cycles (s)	M_s_	M_f_	A_s_	A_f_	H^A–M^ (J/g)	H^M–A^ (J/g)
30	0	15	5	−3	7	4.342	−4.312
30	60	16	6	−4	9	4.001	−3.987
30	150	16	7	−1	10	3.275	−3.128
30	200	17	9	0	11	2.908	−2.897
45	0	15	5	−3	7	4.342	−4.312
45	60	16	3	−5	9	3.765	−3.234
45	150	17	1	−4	12	2.001	−2.289
45	200	20	−5	−9	14	1.621	−1.713
70	0	15	5	−3	7	4.342	−4.312
70	60	18	4	−4	10	2.009	−2.347
70	150	20	3	−2	15	1.512	−1.298
70	200	23	2	1	19	1.110	−1.112

**Table 3:** Transformation temperatures and enthalpies for each heat treatment for 20 min.

Angle	Temperature (°C)	M_s_	M_f_	A_s_	A_f_	H^A–M^ (J/g)	H^M–A^ (J/g)
30	300	-	-	-	-	-	-
30	400	46	26	4	43	4.001	−3.987
30	500	15	3	−3	10	2.178	−2.136
45	300	-	-	-	-	-	-
45	400	37	16	4	33	2.934	−2.923
45	500	16	3	−5	9	4.365	−4.234
70	300						
70	400	28	0	−3	24	1.621	−1.713
70	500	14	6	−2	7	4.333	−4.322

## Data Availability

The authors can provide details of the research requesting by letter and commenting on their needs.
